# Transplantation of Roxadustat‐preconditioned bone marrow stromal cells improves neurological function recovery through enhancing grafted cell survival in ischemic stroke rats

**DOI:** 10.1111/cns.13890

**Published:** 2022-06-13

**Authors:** Jiayu Chen, Xiao Lin, Chaojie Yao, Lebohang Anesu Bingwa, Hao Wang, Zhongxiao Lin, Kunlin Jin, Qichuan Zhuge, Su Yang

**Affiliations:** ^1^ Zhejiang Provincial Key Laboratory of Aging and Neurological Disorder Research, Department of Neurosurgery The First Affiliated Hospital of Wenzhou Medical University Wenzhou China; ^2^ Department of Pharmacology and Neuroscience University of North Texas Health Science Center Fort Worth Texas USA

**Keywords:** autophagy, bone marrow stromal cells, HIF‐1α/BNIP3 signal pathway, stroke

## Abstract

**Aims:**

The therapeutic effect of bone marrow stromal cell (BMSC) transplantation for ischemic stroke is limited by its low survival rate. The purpose of this study was to evaluate whether Roxadustat (FG‐4592) pretreatment could promote the survival rate of grafted BMSCs and improve neurological function deficits in ischemia rats.

**Methods:**

Oxygen–glucose deprivation (OGD) and permanent middle cerebral artery occlusion (pMCAO) were constructed as stroke models in vitro and in vivo. Flow cytometry analysis and expression of Bax and Bcl‐2 were detected to evaluate BMSCs apoptosis. Infarct volume and neurobehavioral score were applied to evaluate functional recovery. Inflammatory cytokine expression, neuronal apoptosis, and microglial M1 polarization were assessed to confirm the enhanced neurological recovery after FG‐4592 pretreatment.

**Results:**

FG‐4592 promoted autophagy level to inhibit OGD‐induced apoptosis through HIF‐1α/BNIP3 pathway. GFP and Ki67 double staining showed an improved survival rate of BMSCs in the FG‐4592 group, whereas infarct volume and neurobehavioral score verified its enhanced neurological recovery activity simultaneously. NeuN and Iba‐1 fluorescence staining showed improved neural survival and decreased microglial activation, along with decreased IL‐1β, IL‐6, and TNF‐α levels through the TLR‐4/NF‐kB pathway.

**Conclusions:**

FG‐4592 pretreated BMSCs improve neurological function recovery after stroke and are likely to be a promising strategy for stroke management.

## Graphical abstract

Oxygen–glucose deprivation or ischemia‐induced apoptosis of BMSCs. FG‐4592‐induced activation of HIF‐1α/BNIP3 signal pathway, and further activation of autophagy to inhibit apoptosis of BMSCs. Eventually, FG‐4592 improves grafted BMSCs survival and enhances therapeutic potential of BMSCs.

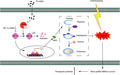

## 1 INTRODUCTION

Ischemic stroke poses a tremendous burden to society with high rates of disability and mortality.^1^ When an ischemic stroke occurs due to cerebral blood flow obstruction, a series of pathophysiological reactions are activated (e.g., inflammatory response, oxidative stress, and cytotoxicity).^2^ Within this process, brain edema develops, which is the consequence of the breakdown of the blood–brain barrier and cytotoxicity, leading to herniation and death.^3^ Another fatal outcome of stroke is hemorrhagic transformation during the reperfusion phase, which increases the risk of neurological deterioration.^4^ Up to now, tissue plasminogen activator (tPA) is the only drug treatment authorized for clinical use.^5^ Regrettably, less than 10% of the patients benefit from tPA administration owing to its strict therapeutic indications.^6^ The most effective timing of tPA administration is within 4.5 h of stroke onset.^7^ Nevertheless, 20%–25% of strokes occur when patients are asleep, so the best therapeutic window may be missed.^8^ Besides, patients with the following special conditions are not recommended for fibrinolysis: more than 80 years of age, baseline NIHSS >25, previous medical history of both stroke and diabetes, and patients taking oral anticoagulants not based on the international normalized ratio (INR).^9^

Mesenchymal stem cell (MSC) transplantation has developed into an encouraging strategy for stroke treatment in recent years.^10^ The prospect of MSCs is clinical application derived from their distinctive characteristics including immune modulation, great paracrine capacity, and multipotent differentiation potential.^11^, ^12^ Many researchers demonstrated that BMSC transplantation could augment angiogenesis and neurogenesis after stroke.^13^ In addition, transplanted BMSCs enhance axonal plasticity, which may improve endogenous neurological restoration. Although accumulating evidence demonstrates that BMSC transplantation has lots of advantages for stroke treatment, a number of problems still limit its therapeutic efficiency. The harsh microenvironment in the ischemic brain such as hypoxia, inflammatory cascade, and formation of reactive oxygen species (ROS) results in poor survival and engraftment of BMSCs after transplantation.^14^ Accordingly, there is an urgent need to raise the survival rates of BMSCs in an ischemic brain.

Hypoxia‐inducible factor 1 alpha (HIF‐1α), a core modulator in hypoxic states, is prone to be inactivated by hypoxia‐inducible factor prolyl hydroxylase (HIF‐PHD) in normoxic states.^15^ There are numerous HIF‐1α target genes involved in pathophysiological processes like angiogenesis, proliferation, metastasis, and invasion.^16^ Previous studies have shown protective effects on ischemic disease including ischemic stroke, heart infarction, and renal injury.^17^, ^18^, ^19^ Moreover, in traumatic brain injury (TBI) rats, neuronal loss was attenuated by HIF‐1α mediated increase of glucose transport activity.^20^ HIF‐1α also attenuates neuronal apoptosis in ischemic stroke.^21^ Roxadustat (FG‐4592) stabilizes the level of HIF‐1α and activates the HIF‐1α‐related pathway.^22^ More importantly, FG‐4592 is used for treating anemia in chronic kidney disease patients with minimal side reactions. Yet whether FG‐4592 preconditioning improves the survival of transplanted BMSCs is unknown. Thus, we explored the effect and possible mechanisms of FG‐4592 in transplanted BMSCs.

## 2 MATERIALS AND METHODS

### 2.1 Procedure of PMCAO model

Adult male Sprague–Dawley rats (230–250 g) were purchased from the Zhejiang Vital River Laboratory Animal Technology Co., Ltd. and transferred to the Animal Laboratory of the First Affiliated Hospital of Wenzhou Medical College. Rats were housed under 12/12 h light/dark cycle condition (room temperature of 20–26°C and relative humidity of 40%–70%), with food and water available ad libitum. PMCAO model was built by blocking the left middle cerebral artery. In short, anesthesia was elicited by inhalation of 5% isoflurane and then maintained at 2% in a mixture of N_2_O:O_2_ (70%:30%). After a longitudinal incision in the midline of the neck, we cautiously separated and ligated the left common carotid artery (CCA), external carotid artery (ECA), and internal carotid artery (ICA). Then, a tiny V‐shape incision was made in ECA by using a microsurgical scissor. A high‐level monofilament (Beijing Sinotech, China) was inserted from the incision to the ICA. When the monofilament had been inserted to about 17.5 mm into the ICA, the outer segment of the thread was fixed. Subsequently, the incision in the neck was sutured. During surgery, a heating pad was used to maintain the core body temperature of the rats within 36–37°C until they recovered from anesthesia, and then, the rats were transferred to their cage on a heating pad for 12 h to maintain body temperature at 36–37°C. Apart from inserting the thread, the sham group received all the operations. All animal experiments were approved by the Animal Care Committee of Wenzhou Medical University (China), and all animal procedures were in compliance with the NIH guidelines for animal use. The animal data reporting has followed the ARRIVE 2.0 guidelines.^23^

Rats were randomly divided into the following groups by an investigator who is blinded to group assignment: sham group, pMCAO group (rats were subjected to pMCAO surgery only), BMSC group (rats were subjected to pMCAO surgery and received BMSC transplantation), and F‐BMSC group (rats were subjected to pMCAO surgery and received FG‐4592 pretreated‐BMSC transplantation).

### 2.2 Cell culture and transplantation

Bone marrow stromal cells were extracted from the bone marrow of SD rats as previously mentioned,^24^ and cultured in the complete medium, a mixture of 89% Dulbecco's modified Eagle's medium (DMEM; Gibco), 10% fetal bovine serum (FBS; Gibco), and 1% penicillin (100 U/ml)/streptomycin (100 μg/ml) (Gibco). To identify the cell phenotype, flow cytometry (CytoFLEX, Beckman Coulter) was used to detect cell surface antigens (CD29, CD44, CD90, CD45, CD34, and HLA‐DR). The differentiation potential of BMSCs toward chondrocyte, osteocytes, and adipocytes was investigated through culturing in a corresponding differentiation medium (iCell Bioscience Inc.). Before transplantation, the lentiviral vector was applied to establish GFP‐BMSCs. For each GFP‐BMSC transplantation treatment, 5 × 10^5^ cells in 4 μl of DMEM medium were injected (1 μl/min) into the peri‐infarction area with a micro‐injection needle (Hamilton 7000) 1 day after stroke onset. In the F‐BMSC group, BMSCs were treated with FG‐4592 (10 μmol/L; Cat. No.: GC13139, GLPBIO) for 24 h before transplantation. The other groups only received saline injections.

### 2.3 Oxygen–glucose deprivation

Briefly, BMSCs were first cultured in a glucose‐free DMEM (Gibco) in an anaerobic incubator (Heal Force) and flushed with CO_2_ (5%), O_2_ (1%) and N_2_ (94%) for 24 h at 37°C. The normal medium under normoxia severed as the control. FG‐4592 (10 μmol/L) was given prior to the oxygen–glucose deprivation (OGD) insult.

### 2.4 Small interfering RNA transfection

For HIF‐1α silencing, small interfering RNA (siRNA)‐HIF‐1α, and riboFECT CP reagents (cat. no. C10511‐1, Guangzhou RiboBio Co., Ltd.) were used for the transfection of BMSCs. Firstly, BMSCs were incubated in 6‐well plates with 1 × 10^6^ cells/well and followed by blending siRNA‐HIF‐1α (5 μl; three targeted sequences: siRNA‐HIF‐1α‐001, CTGATAACGTGAACAAATA; siRNA‐HIF‐1α‐002, TCGACAAGCTTAAGAAAGA; siRNA‐HIF‐1α‐003, GGACAATATAGAAGACATT) into 1× riboFECT CP buffer and riboFECT CP reagent to form a transfection mixture. BMSCs were then transfected with the mixture and cultured in DMEM for 72 h. NC siRNA (targeted sequence: GGCTCTAGAAAAGCCTATGC) was used for the negative control. Based on western blot analysis, the most effective sequence of siRNA‐HIF‐1α was selected for subsequent experiments.

### 2.5 Cell counting kit‐8 assay

For the cell counting kit‐8 (CCK‐8) test, BMSCs were cultured with different concentrations of FG‐4592 for 24 h, followed by OGD exposure. Subsequently, each well received 10‐μl CCK‐8 reagent (Dojindo), and optical density (OD) values were obtained using a SpectraMax 190 (Molecular Devices) after 2 h at a wavelength of 450 nm.

### 2.6 Ki67 immunofluorescence staining

The proliferation of BMSCs post‐OGD was measured by Ki67 staining. Firstly, BMSCs were fixed in 4% paraformaldehyde (Beyotime Institute of Biotechnology) for 30 min and permeabilized for 20 min in 0.2% Triton X‐100 (Beyotime Institute of Biotechnology). Then, BMSCs were blocked with 5% bovine serum albumin solution (BSA; BioFroxx) for 2 h. Anti‐Ki67 antibody (Abcam, ab15580, 1:1000) was used to incubate BMSCs at 4° C overnight. After washing three times, BMSCs were incubated with goat anti‐rabbit IgG (H + L) DyLight 488 secondary antibody (Abcam, ab150077, 1:1000). DAPI (Thermo Fisher Scientific) was used for nuclei staining. Five random fields per section were captured with a fluorescence microscope (Leica Microsystems DMi8).

### 2.7 Flow cytometry for apoptosis

After OGD exposure, BMSCs were digested and centrifuged. Then, cell pellets were incubated with 195‐μl Annexin V‐FITC binding solution with 10‐μl propidium iodide (PI) and 5‐μl Annexin V‐FITC (Beyotime Institute of Biotechnology) for 20 min. 4 × 10^5^ cells in each group were assayed by flow cytometry within 1 h, and then, results were analyzed by CytExpert software (Beckman Coulter).

### 2.8 Analysis of western blot

Samples were collected and lysed in the radioimmunoprecipitation assay buffer (RIPA; Thermo Fisher) supplemented with phenylmethanesulfonyl fluoride (PMSF) for 0.5 h on ice. Sample concentrations were determined by the BCA protein assay kit (Thermo Fisher). The proteins were run on SDS‐PAGE gels (8%–12%) and then transferred to polyvinylidene difluoride (PVDF) membranes (Millipore). Each PVDF membrane was incubated with 5% BSA for 2 h. After being incubated with the corresponding primary antibodies (HIF‐1α [Abcam, ab179483, 1:1000], LC3B [Abcam, ab192890, 1:1000], P62 [Affinity, AF5384, 1:1000], Beclin‐1 [Affinity, AF5128, 1:1000], Bcl‐2 [Affinity, AF6139, 1:1000], Bax [Affinity, AF0120, 1:1000], GAPDH [Affinity, AF7021, 1:1000], BNIP3 [Affinity, DF8188, 1:1000], NF‐kB [Affinity, AF5006, 1:1000], and TLR4 [Proteintech, 19,811‐1‐AP, 1:1000]) at 4°C overnight, membranes were washed three times with TBST (TBS with 0.1% Tween 20) and further incubated with secondary antibodies (HRP‐conjugated Affinipure Goat Anti‐Rabbit IgG [H + L] [Proteintech, SA00001‐2, 1:10,000]) for 2 h. Protein blots were exposed with a ChemiDoc XRS apparatus (Bio‐Rad Laboratories, Inc.).

### 2.9 Cerebral infarct volume assessment

The complete rat brain was separated immediately after euthanasia 3 days post‐transplantation. The whole brain was cut into five serial 2‐mm‐thick slices using a standardized rodent brain matrix. Each slice was immersed in 2.0% TTC solution (Beijing Solarbio Science & Technology Co., Ltd.) at 37°C for 0.5 h and kept away from light. The normal area would be red, whereas the infarcted tissue would be white. The extent of infarction was analyzed by ImageJ software (National Institutes of Health). The ischemic area was calculated according to the following formula: Infarct volume = (contralateral hemisphere region—non‐infarcted region in the ipsilateral hemisphere) × 2‐mm thickness. Data were presented as absolute numbers.

### 2.10 Immunofluorescence assays

Rats were anesthetized and transcardially perfused with 4% paraformaldehyde. The whole brain was then isolated, followed by fixing in 4% paraformaldehyde at 4°C overnight. Before fabricating frozen sections, the tissues were immersed in 30% sucrose until thoroughly dehydrated. Coronal brain sections located between +1.5 and ‐3 mm from the bregma were used for staining. And, brain sections in the same location were selected for immunocytochemistry The sections (10 μm) were treated with antigen retrieval buffer (Beyotime Institute of Biotechnology) and blocked in 5% BSA with 0.2% TritonX‐100 in PBS (Gibco) for 2 h. Subsequently, each section was incubated with the primary antibodies (Iba1 [1:200, Abcam, ab5076], GFP [1:1000, Abcam, ab13907]) at 4°C overnight, followed by incubation with corresponding secondary antibodies in the dark. Finally, the nuclei were labeled with DAPI. Micrographs were photographed from the ischemic penumbra (Figure 4C) with a scanning fluorescence microscope at 20× magnification. The parameters of the microscope, including exposure time, gain compensation, and aperture size, were consistent during the photographic process. Five random microscopic fields from each section were taken for analysis. Quantified analysis was performed with ImageJ software. The data obtained from five fields of each section were averaged as one value. All histological assessments were conducted by researchers who were blinded to the experimental design.

### 2.11 Apoptotic neuron staining

Apoptotic neurons were assessed by NeuN and TUNEL double staining. After finished fixation, permeation, and blocking, samples were incubated with primary antibody (NeuN, 1:1000, Abcam, ab177487) and corresponding secondary antibody to mark neurons. Then, samples were washed three times, followed by further incubation with a TUNEL reaction kit (Beyotime Institute of Biotechnology) for 1 h at 37°C in a darkroom. DAPI was employed for nuclear staining and observed under a fluorescence microscope. Apoptotic neurons were assessed in five randomly selected fields from each section.

### 2.12 Neurobehavioral evaluations and body weight measurement

Neurobehavioral evaluations were measured by a modified neurological severity score (mNSS) 1 day before the stroke, 0, 3, 7, 14, and 28 days after the stroke. Rats were trained for the test before the stroke. The points of mNSS included motor, sensory, reflex, and balance scores, with a maximum score of 18. Higher scores corresponded with severe neurological injury. Meanwhile, rats were weighed at 0, 1, 3, 7, 14, and 28 days after stroke.

The number of rats which received MCAO surgery is 63, and then, the rats were randomly divided into three groups (pMCAO group, BMSC group, and F‐BMSC group) for behavior test. At 28 days after MCAO surgery, the number of rats in three groups was 12, 13, and 15, respectively. In order to ensure consistency between groups, 12 rats were selected from the BMSC group and F‐BMSC group, respectively, according to the randomized and blinded principle. All investigators involved in the evaluation process were blinded to the experimental groups.

### 2.13 Enzyme‐linked immunosorbent assay

Serum was extracted from whole blood by centrifuging at 1500 *g* for 10 min. Then, levels of interleukin (IL)‐6, IL‐1β, and tumor necrosis factor (TNF)‐а in serum were measured using respective enzyme‐linked immunosorbent assay (ELISA) kits (E‐EL‐R0015c, E‐EL‐R0012c, E‐EL‐R2856c; Elabscience Biotechnology) based on the manufacturer's instructions. Absorbance values at 450 nm were detected using a microplate reader.

### 2.14 Statistical analysis

All data were expressed as average ± standard deviation (SD). The normality of all data was tested by the Shapiro–Wilk test, and the results showed that all data were normally distributed. Statistical differences were valued by t‐test, one‐way, or two‐way analysis of variance (ANOVA) using GraphPad Prism 5. All comparisons would be regarded as statistically significant, while *p* value <0.05.

## 3 RESULTS

### 3.1 Pretreatment of FG‐4592 improves viability and proliferation of BMSCs after OGD

**FIGURE 1:**
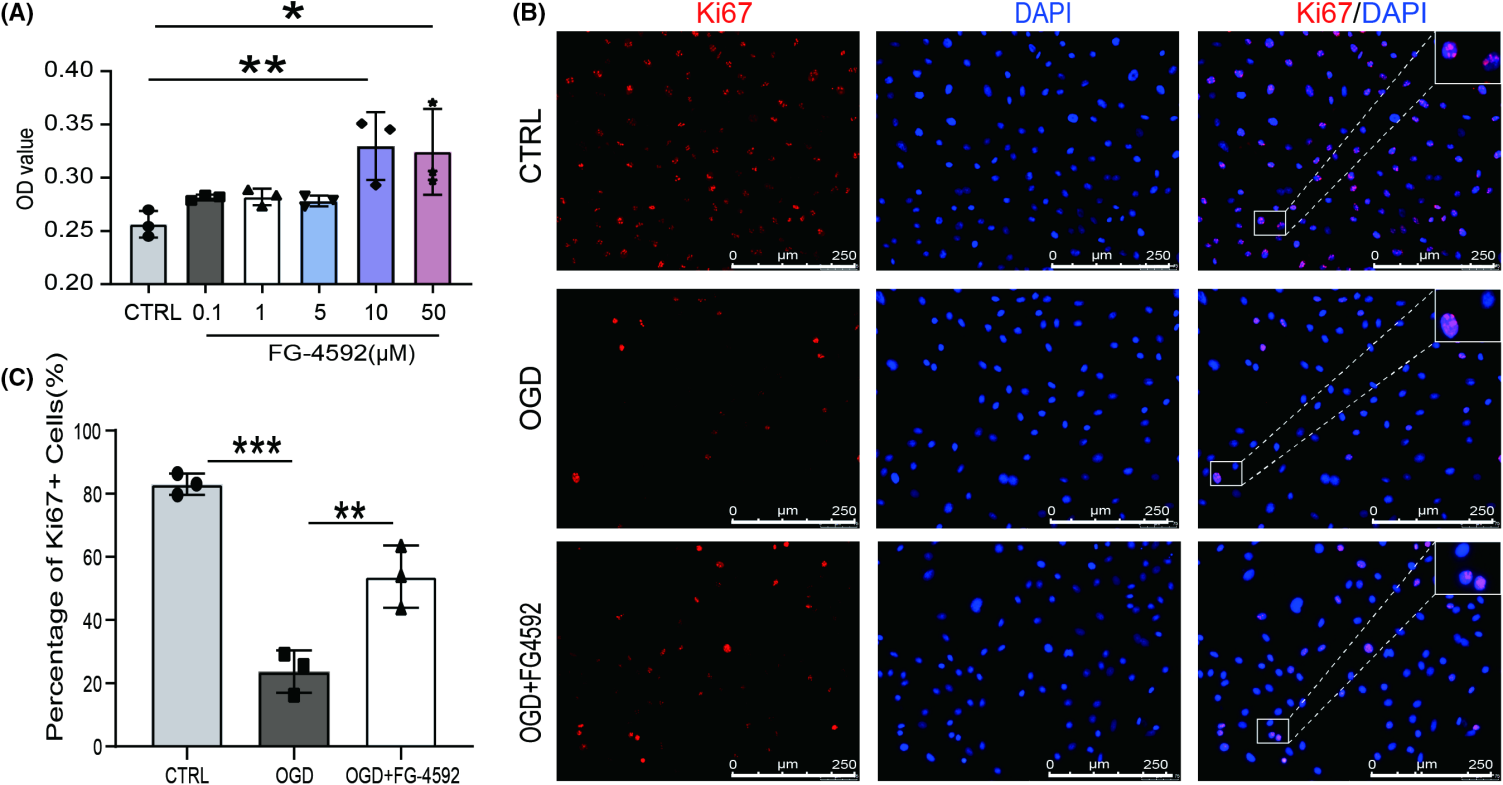
FG‐4592 improves viability and proliferation of bone marrow stromal cell (BMSCs) after oxygen–glucose deprivation (OGD). (A) The effect of FG‐4592 on the viability of BMSCs post‐OGD. (B–C) Proliferation of BMSCs post‐OGD was analyzed by Ki67 staining, scale bars = 250 μm. For each group in every experiment: *n* = 3; * means *p* value <0.05, ** means *p* value <0.01, and *** means *p* value <0.001, *p* values were calculated with one‐way ANOVA, followed by Bonferroni's multiple comparison test

Bone marrow stromal cells were identified by surface antigens and multi‐directional differentiation potential through alizarin red, Alcian blue, and oil red O staining (iCell Bioscience Inc.), respectively (Figure S1). After that, the role of FG‐4592 pretreatment in BMSCs under OGD exposure was evaluated. CCK‐8 assay showed that the viability of BMSCs, which were pretreated with 10 μmol/L of FG‐4592 for 24 h, improved significantly (Figure 1A). Immunofluorescent staining with Ki67 following OGD exposure showed decreased expression of Ki67, while FG‐4592 pretreatment increased the quantity of Ki67 positive cells (Figure 1B,C). These results indicated that FG‐4592 enhanced the survival ability of BMSCs under OGD exposure.

### 3.2 FG‐4592 protects BMSCs from OGD‐induced apoptosis and promotes cellular autophagy through the HIF‐1α‐BNIP3 pathway

**FIGURE 2:**
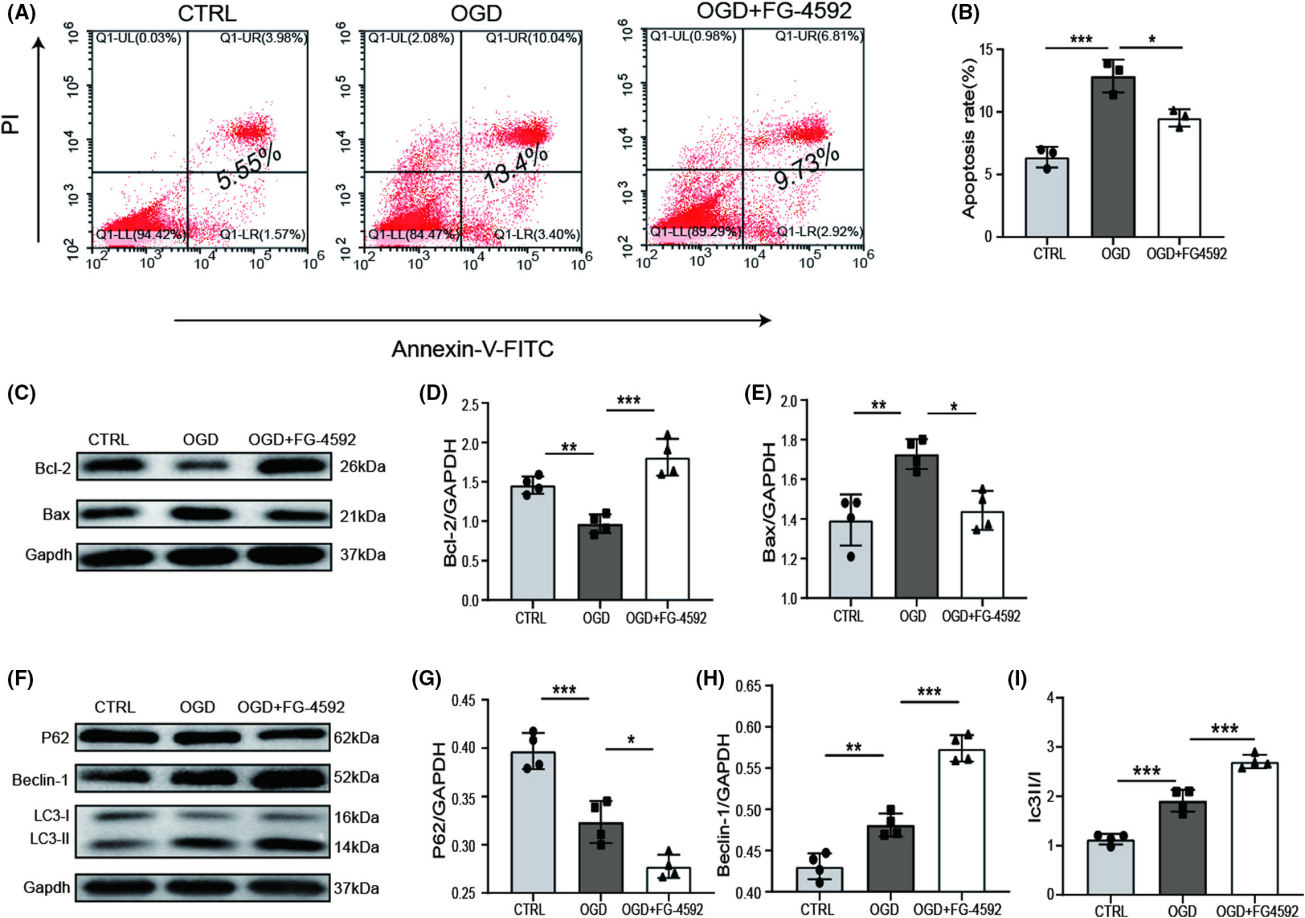
FG‐4592 inhibits apoptosis and promotes autophagy in bone marrow stromal cell (BMSC) post‐oxygen–glucose deprivation (OGD). (A, B) Apoptosis of BMSCs in different groups was stained by Annexin V‐FITC/PI, and then assessed by flow cytometry (FCM). (C–E) Western blot images and relative quantification of apoptosis‐related proteins (Bcl‐2, Bax) and Gapdh (*n* = 4). (F–I) Western blot analysis of P62, Beclin‐1, LC3, and Gapdh and quantitative analysis (*n* = 4). * means *p* value <0.05, ** means *p* value <0.01, and *** means *p* value <0.001, *p* values were calculated with one‐way ANOVA, followed by Bonferroni's multiple comparison test

**FIGURE 3:**
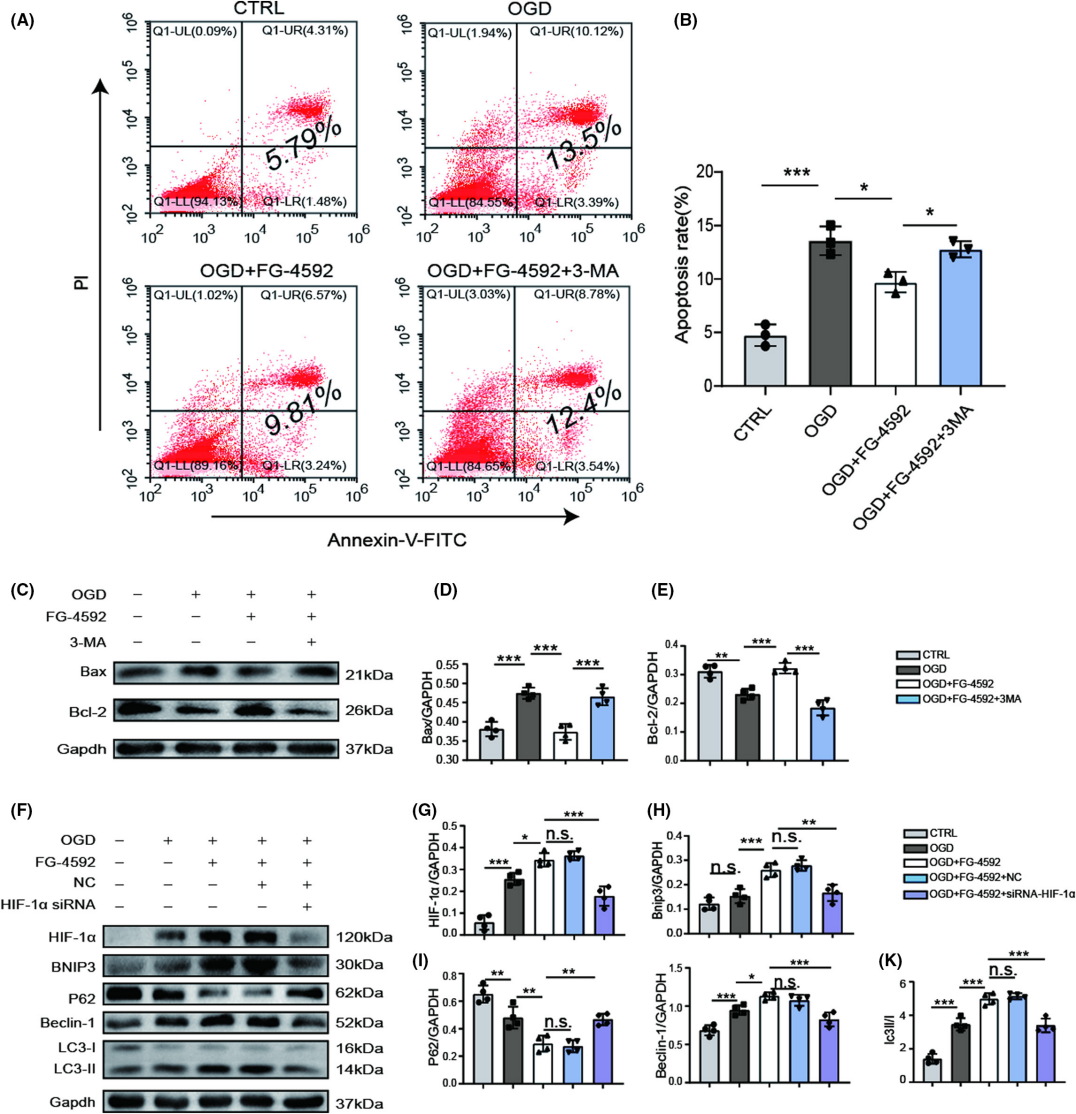
FG‐4592 inhibits apoptosis in bone marrow stromal cell (BMSC) post‐oxygen–glucose deprivation (OGD) through activating HIF‐1α/BNIP3‐dependent autophagy. (A, B) Apoptosis measured by flow cytometry (FCM), and statistics were displayed as mean ± SD. (C–E) Western blot images and relative quantification of apoptosis‐related proteins (Bcl‐2, Bax) and Gapdh (*n* = 4). (F–K) Expression of HIF‐1α, BNIP3, P62, Beclin‐1, LC3, and Gapdh in BMSCs was detected by western blot. And relative quantification of HIF‐1α, BNIP3, P62, Beclin‐1, LC3 (*n* = 4). * means *p* value <0.05, ** means *p* value <0.01, and *** means *p* value <0.001; n.s. means no significance, *p* values were calculated with one‐way ANOVA, followed by Bonferroni's multiple comparison test

We further explored the effect of FG‐4592 against OGD‐induced apoptosis in BMSCs. Through flow cytometry assay, we found that the apoptosis ratio of BMSCs was distinctly increased in the OGD group, whereas FG‐4592 administration decreased the apoptosis rate in BMSCs after OGD (Figure 2A,B). Similarly, pretreatment with FG‐4592 notably suppressed the level of Bax and restored the level of Bcl‐2 (Figure 2C–E), indicating the anti‐apoptosis activity of FG‐4592 in OGD‐treated BMSCs. Autophagy is essential for keeping intracellular homeostasis by degrading damaged proteins and recycling cellular components under stressful conditions.^25^ Here, we observed increased expression of Beclin‐1 and LC3BII/LC3BI ratio, along with reduced expression of P62 in the FG‐4592 pretreatment group (Figure 2F–I). Through inhibiting autophagy using 3‐Methyladenine (3‐MA; 5 mmol/L; Sigma), we found that 3‐MA significantly reverses the anti‐apoptotic effect of FG‐4592 (Figure 3A–E), suggesting FG‐4592 facilitates autophagy to exert its anti‐apoptotic effect against OGD caused damage.

Considering that FG‐4592 serves as a potent activator of HIF‐1α, we then blocked HIF‐1α expression through siRNA interference to investigate the potential mechanism of FG‐4592 in autophagy. After transfecting BMSCs with siRNA‐HIF‐1α, the expression of HIF‐1α was significantly decreased and the autophagy facilitation effect of FG‐4592 was attenuated; meanwhile, the expression of BNIP3, which has been proven to facilitate autophagy,^26^ was reduced visibly (Figure 3F–K). These results indicated that FG‐4592 inhibits apoptosis by regulating autophagy by the HIF‐1α‐BNIP3 pathway.

### 3.3 FG‐4592 pretreatment increases BMSCs survival and improves neurological function recovery in stroke rats

**FIGURE 4:**
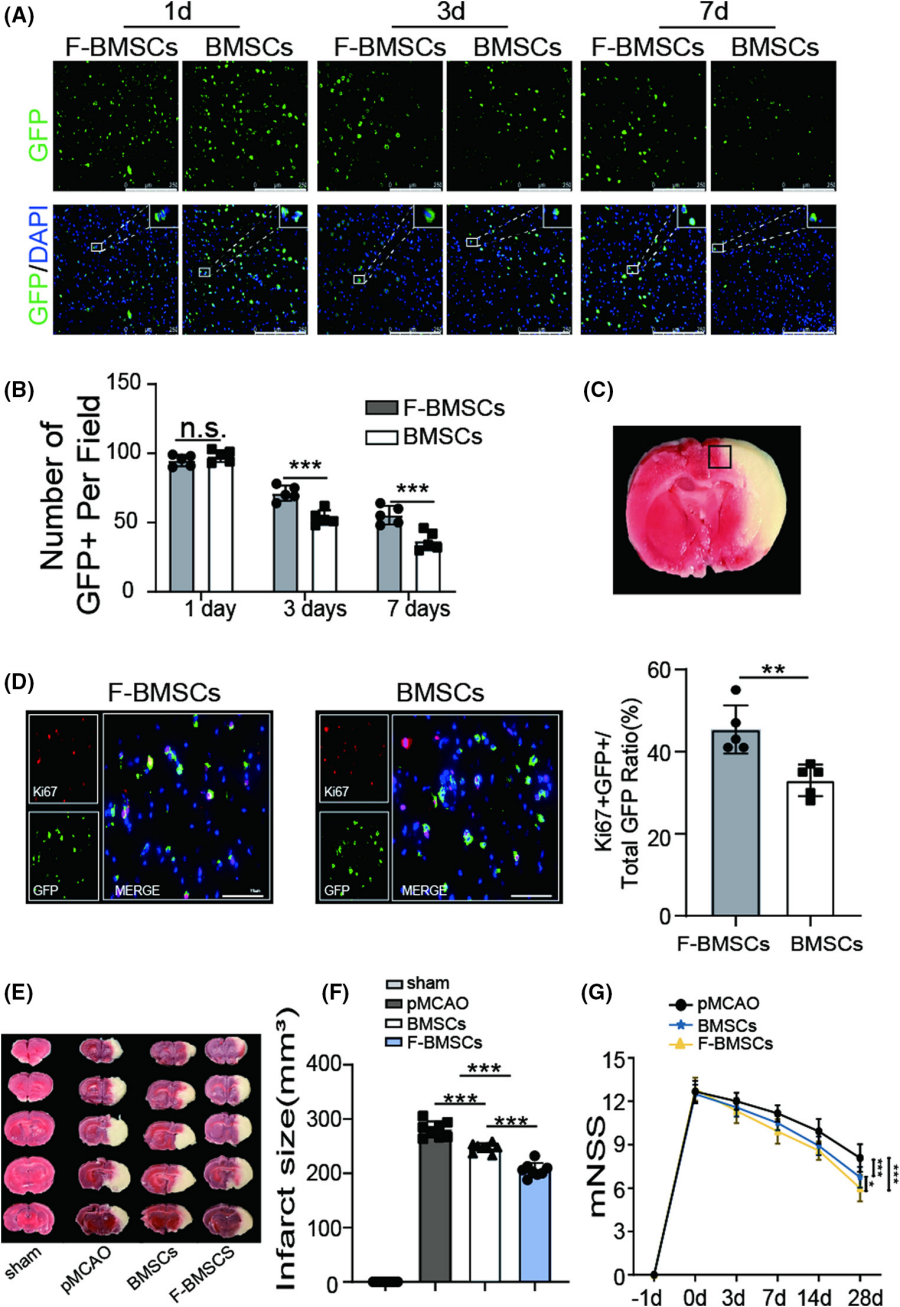
FG‐4592 preconditioning improves bone marrow stromal cell (BMSC) survival in the ischemic brain and facilitates functional recovery. (A, B) Representative images of GFP (green) stained BMSCs in each group at 1, 3, and 7 days after transplantation as shown above, the statistical data were also presented (*n* = 5), *p* values were calculated with two‐way ANOVA, followed by Bonferroni's multiple comparison test. Scale bars = 250 μm (C) Images were captured from the ischemic penumbra. (D) The proliferation of grafted BMSCs was evaluated by Ki67 and GFP double staining (*n* = 5), data were analyzed using two‐tailed *t*‐test, scale bars = 75 μm. (E, F) Infarct areas were visualized by TTC buffer at 3 days post‐PMCAO, statistics were presented as mean ± SD, *p* values were calculated with one‐way ANOVA, followed by Bonferroni's multiple comparison test (*n* = 8). (G) Results of the mNSS of the rat in each group were recorded at −1, 0, 3, 7, 14, and 28 days since stroke onset (*n* = 12), data were analyzed using two‐way ANOVA, followed by Bonferroni's multiple comparison test; * means *p* value <0.05, ** means *p* value <0.01, and *** means *p* value <0.001; n.s. means no significance

Before transplantation, BMSCs were labeled with GFP to determine cell survival and distribution within the brain. The transfection efficacy was identified by fluorescence staining with GFP antibody at 3 days post‐transfection (Figure S2). GFP staining indicated that the quantity of BMSCs in the F‐BMSC group after transplantation was approximately 1.3‐folds on day 3 and 1.5‐folds on day 7 compared with transplantation of BMSCs alone (Figure 4A–C), suggesting FG‐4592 pretreatment increased the survival rate of transplanted BMSCs. In order to detect the proliferation of grafted BMSCs, Ki67 and GFP double staining was performed. The results revealed that FG‐4592 significantly improves the proliferation of grafted BMSCs (Figure 4D). After that, we used TTC staining to evaluate the ischemic infarct focal area. The result showed that massive hemispheric infarction (280.9±14.8 mm^3^) was caused by occlusion of the middle cerebral artery, and BMSC transplantation effectively reduced the infarct area, approximately 247.2±8.3 mm^3^. More importantly, transplantation of FG‐4592‐pretreated BMSCs further reduced infarct size to 206.2±12.9 mm^3^ (Figure 4E–F). mNSS showed that the BMSC group and F‐BMSC group had lower mNSS than the pMCAO group. At 28 days after stroke, the score of the BMSC group was 6.75 ± 0.72, which was significantly lower than that of the pMCAO group, approximately 8.08 ± 0.95, whereas the score of the F‐BMSC group was even lower at 6 ± 0.91, which was more significant than that of BMSC group (Figure 4G), indicating FG‐4592 facilitates the therapeutical effect of BMSCs in stroke rats. Before surgery, the average body weight of all groups was identical. Rats in all groups had a reduction in body weight during the initial first few days after surgery, but afterward, body weight was increased. There were no significant differences at day 28 among pMCAO groups, BMSC group, and F‐BMSC group (Figure S3).

### 3.4 FG‐4592‐pretreated BMSCs prevent neuronal apoptosis in stroke rats

**FIGURE 5:**
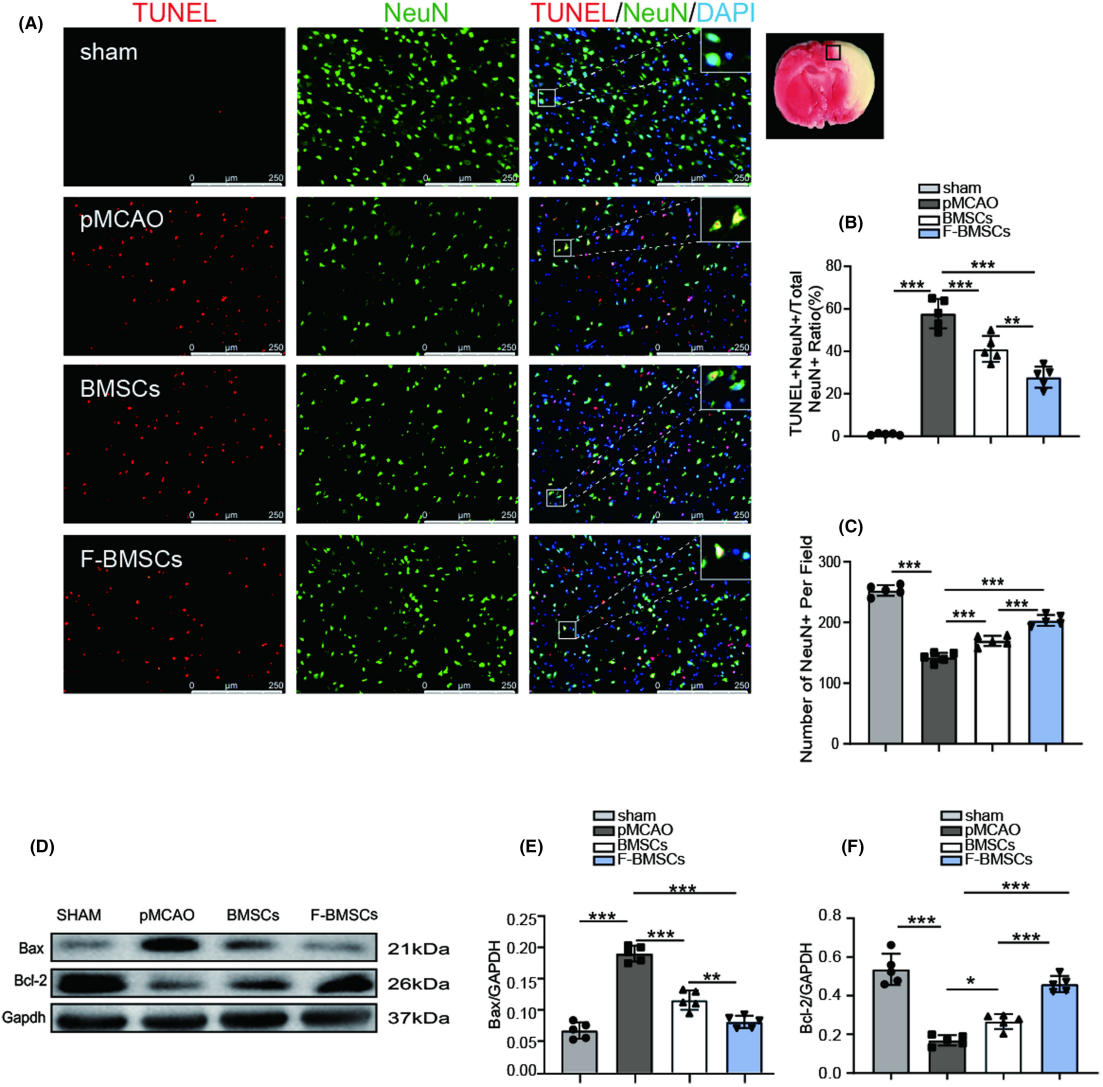
Transplantation of FG‐4592‐preconditioned bone marrow stromal cell (BMSCs) improves neuronal apoptosis after stroke. (A) Representative images of TUNEL and NeuN co‐staining in different groups, scale bars = 250 μm. (B) Quantification analysis of neuronal apoptotic rate in every group (*n* = 5). (C) Quantitative calculation of surviving neurons in each group (*n* = 5). (D–F) Western blot images of Bcl‐2, Bax, and Gapdh and relative quantitative analysis (*n* = 5); * means *p* value <0.05, ** means *p* value <0.01, and *** means *p* value <0.001, *p* values were calculated with one‐way ANOVA, followed by Bonferroni's multiple comparison test

To test whether transplantation of FG‐4592‐pretreated BMSC could reduce neuronal apoptosis after stroke, NeuN and TUNEL double staining was performed. As shown in Figure 5A, we discovered that BMSC transplantation reduced the proportion of apoptotic neurons in the ischemic penumbra, from 57.8 ± 6.86% to 41.1 ± 6.13%. More importantly, compared with BMSC transplantation alone, fewer TUNEL positive neurons were found in the F‐BMSC group (Figure 5B), with a ratio of 27.9 ± 4.99%. The number of neurons was significantly increased after implantation of FG‐4592‐pretreated BMSCs or BMSCs alone. Moreover, FG‐4592‐pretreated BMSC transplantation further increased the NeuN‐positive cell number compared to the BMSC group (Figure 5C). Besides, results of immunoblotting revealed significantly higher expression of Bcl‐2 and much less expression of Bax in the F‐BMSC group compared with the BMSC group (Figure 5D–F). These findings demonstrated that FG4592‐pretreated BMSCs possess anti‐apoptotic properties in ischemic stroke rat models.

### 3.5 FG‐4592‐pretreated BMSCs inhibit microglial activation and inflammation through TLR4/NF‐kB pathway after stroke

**FIGURE 6:**
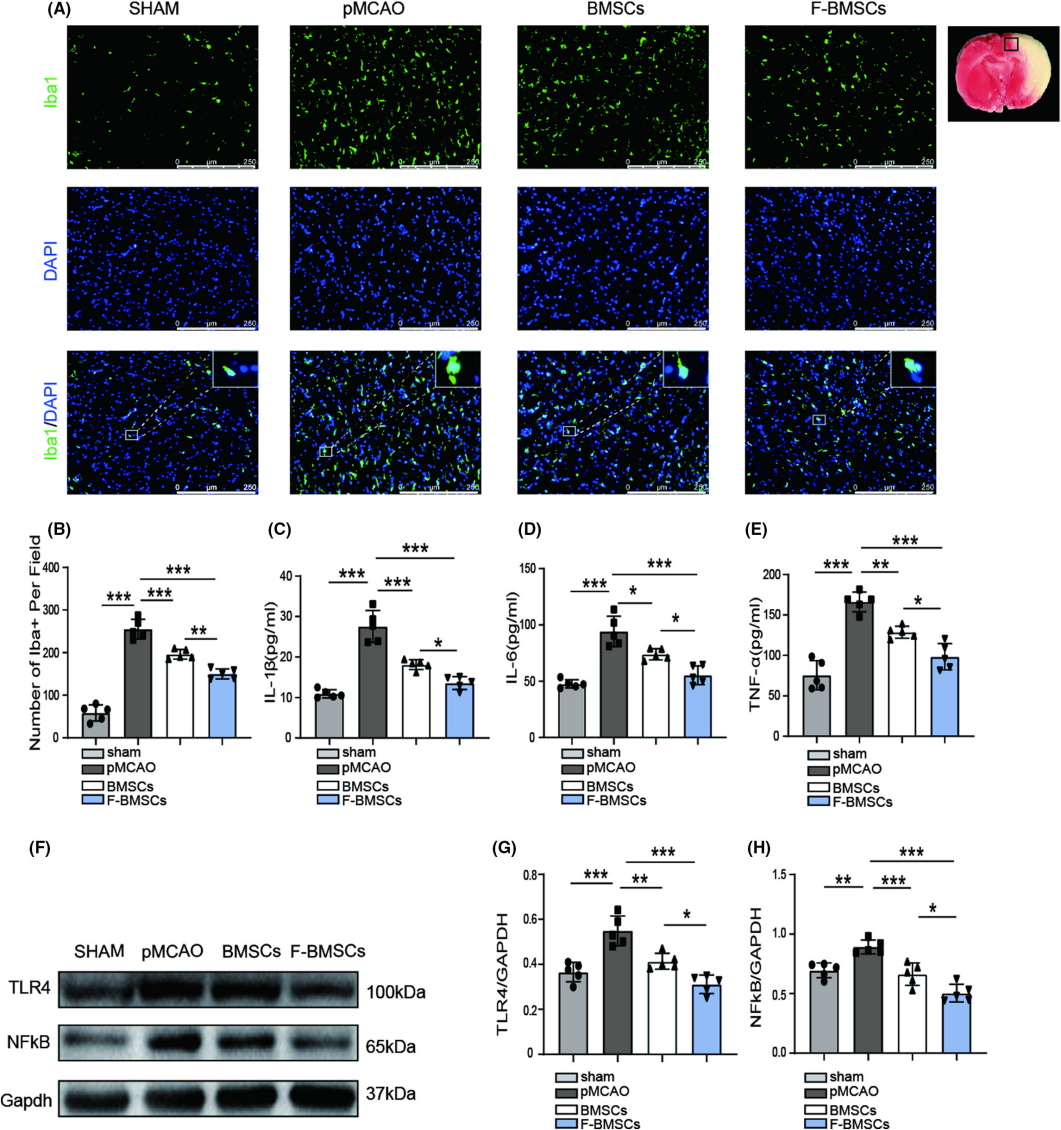
Transplantation of FG‐4592 preconditioned bone marrow stromal cell (BMSCs) inhibits microglial activation and inflammation. (A, B) Representative images and quantitative analysis of Iba1 staining (*n* = 5), scale bars = 250 μm. (C–E) Quantification of IL‐1β, IL‐6, and TNF‐α in each rat was assessed by ELISA (*n* = 5) (F–H) Western blot images and relative quantitative analysis of TLR4, NF‐kB, and Gapdh (*n* = 5); * means *p* value <0.05, ** means *p* value <0.01, and *** means *p* value <0.001, *p* values were calculated with one‐way ANOVA, followed by Bonferroni's multiple comparison test

Microglia are important in maintaining brain microenvironment homeostasis, and to a large extent, microglial activation contributes to the inflammatory response in the setting of stroke.^27^ Therefore, we stained Iba1 at the peri‐infarction area to explore microglial activation. Both the BMSC group and F‐BMSC group notably reduced the Iba1‐positive cell number, with greater reduction observed in the F‐BMSC group (Figure 6A,B). In addition, ELISA showed that levels of pro‐inflammatory factors (IL‐1β, IL‐6, TNF‐α) were increased after ischemic stroke. As predicted, BMSC transplantation reduced the level of these factors. Moreover, IL‐1β, IL‐6, and TNF‐α were further reduced in the F‐BMSC group in comparison with the BMSC group (Figure 6C–E). Toll‐like receptor 4 (TLR4), a single transmembrane cell‐surface receptor that is involved in innate immune response, has a critical role in the activation of microglia.^28^, ^29^ As one of the downstream molecules of TLR4, NF‐kB is an inflammatory transcription factor and is required for the release of IL‐1β, IL‐6, TNF‐α.^30^ Therefore, TLR4/NF‐kB pathway in the ischemic stroke rat models after transplantation was investigated. Treatment with BMSCs markedly reduced the levels of TLR4 and NF‐kB (Figure 6F). The expression of TLR4 and NF‐kB was further reduced after FG‐4592‐pretreated BMSC transplantation compared with BMSCs alone (Figure 6F–H). These data imply that FG‐4592‐pretreated BMSCs inhibited microglial activation and inflammation through TLR4/NF‐kB pathway.

## 4 DISCUSSION

To handle the predicament, which is the poor survival rate of BMSCs after transplantation in ischemic stroke rats, great emphasis was paid on improving the harsh microenvironment in and around the infarct zone. Jiangnan Hu and colleagues discovered that the administration of a calpain inhibitor, MDL28170, at 30 min post‐TBI attenuated the release of inflammatory factors so that more BMSCs survived after transplantation.^24^ Meanwhile, several triggers or genetic modifications were also investigated to enhance tolerance of grafted BMSCs to critical living environments. Transplantation of nerve growth factor (NGF) and Noggin overexpressed BMSCs significantly facilitated the survival of BMSCs in stroke.^31^ Wu et al. showed that NT3P75–2 gene‐modified BMSCs inhibit neuroinflammation and ameliorate neurological function deficits through elevating survival rates of BMSCs in a TBI model.^32^ In this study, the function of FG‐4592 in BMSCs‐implanted stroke models in vitro and in vivo was investigated. The consequences demonstrated that FG‐4592‐pretreated BMSCs have higher survival rates and more potent effects on improving neurological recovery than BMSCs alone in stroke.

Under ischemic or hypoxic conditions, apoptosis of BMSCs was induced by a lack of ATP or insults secondary to extracellular stress.^33^ In our study, OGD‐induced apoptosis of BMSCs was inhibited following FG‐4592 pretreatment. A recent study showed that autophagy, by way of innate defense mechanisms, was activated in Neuro‐2a cells under OGD, a finding consistent with our observations.^34^ The expression of autophagy‐related proteins revealed that autophagy was induced after FG‐4592 pretreatment. Therefore, the anti‐apoptotic effects of FG‐4592 might be mediated by autophagy activation. It is known that autophagy serves as a guardian of cellular homeostasis because of the effect of degrading cytotoxic products.^35^ During hypoxia, cells suffer mitochondrial damage, leading to the opening of mitochondrial permeability transition pores (mPTP) and the release of pro‐apoptotic factors which finally mediate cell death.^36^ On the other hand, when cells receive autophagy‐inducing molecular signals, abnormal proteins and damaged organelles are incorporated into autophagosomes, followed by degradation via the lysosomal pathway. We then explored whether suppression of autophagocytosis enhances apoptosis using 3‐MA. As predicted, 3‐MA attenuated FG‐4592‐induced anti‐apoptotic effects on BMSCs post‐OGD.

In addition, FG‐4592 inhibits prolyl hydroxylases (PHD) such that HIF‐1α is activated under normoxic conditions.^37^ HIF‐1α signal pathway has already been proven to be associated with autophagy. For example, granulosa cell luteinization was enabled by HIF‐1α/BNIP3‐mediated autophagy which also protected granulosa‐lutein cells against hypoxia.^38^ Moreover, BNIP3 is a downstream protein of HIF‐1α closely related to autophagy.^39^ The way BNIP3 regulates autophagy is activating mitophagy or detaching Bcl‐2‐Beclin1 complex to activate autophagy.^40^, ^41^ Hence, we further investigated the regulatory mechanism of FG‐4592 in autophagy by BMSCs transfected with HIF‐1α siRNA. Our results showed that FG‐4592 activated autophagy through HIF‐1α/BNIP3 signaling pathway.

The therapeutic mechanisms of BMSCs in stroke mainly include immunomodulation, improving angiogenesis and neurogenesis, and release of trophic factors and exosomes.^42^, ^43^, ^44^ BMSCs suppress inflammatory response and increase the generation of insulin‐like growth factor 1 (IGF‐1) and vascular endothelial growth factor (VEGF), further inhibiting neuronal apoptosis which is consistent with our results.^45^, ^46^ Moreover, numerous evidence has demonstrated that HIF‐1α improves the production of VEGF, IGF‐1, and EPO which all possess neuroprotective effects of BMSCs.^47^, ^48^ More importantly, BMSCs originally exist in the low oxygen concentration bone marrow niche, and HIF‐1α probably is essential for the migration, proliferation, and differentiation of BMSCs.^49^ In our study, increased survival of grafted BMSCs and significant improvement of functional outcome was found after transplantation of FG‐4592‐pretreated BMSCs compared with BMSCs only.

Following stroke, microglial polarization is responsible for the inflammatory responses with the release of inflammatory mediators.^50^ Sun and colleagues indicated that preconditioning with isoflurane alleviates ischemic damage by inhibition of microglial activation in stroke rats.^51^ Herein, microglial activation was attenuated as shown by Iba1 staining. Consistent with previous reports,^52^ BMSCs reduced inflammatory mediators in our study, as measured by ELISA. Furthermore, these neuroprotective, anti‐inflammatory effects were augmented by pretreatment with FG‐4592. On the other hand, a growing number of researchers have suggested that FG‐4592 could hold neuroprotective effects in some central nervous system disorders. For instance, treatment with FG‐4592 could improve neurological recovery in spinal cord injury.^53^ Gaifen Li et al. found that the administration of FG‐4592 facilitated neurogenesis and synaptic plasticity, thereby attenuating memory impairment and depression.^54^ All this evidence confirms that FG‐4592 can serve as a favorable preconditioning trigger for stem cell‐based therapy.

Our present work fully demonstrates that FG‐4592 reinforces neuroprotective effects in BMSC transplanted stroke rats, but further developments are needed before consideration as a potential clinical therapeutic. In addition, it is important to investigate the number, migration, and differentiation of transplanted BMSCs in long‐term experiments. The secretome of FG‐4592‐preconditioned BMSCs also needs to be assessed to clearly clarify its neuroprotective mechanisms. Last but not least, larger cohorts and more detailed experiments are indispensable in verifying the safety of BMSC transplantation.

## 5 CONCLUSION

Overall, the present work demonstrates that FG‐4592 protects BMSCs from OGD by activating autophagy, which facilitates the survival of grafted BMSCs in the ischemic microenvironment (Figure S4). Besides, FG‐4592‐pretreated BMSCs further ameliorate inflammation and apoptosis, leading to an improvement in the neurological function of ischemic stroke rats. These discoveries suggest a novel approach for boosting the effectiveness of stem cell therapy in ischemic stroke.

## AUTHOR CONTRIBUTIONS

SY, QCZG, and KLJ designed the study and provided the critical effect. JYC and XL performed the main experiments. CJY, HW, and ZXL analyzed the data and performed the statistical analyses. SY, JYC, and QCZG edited the manuscript. JYC, XL, and LB revised the manuscript. All authors gave feedback and agreed on the final version of the manuscript.

## CONFLICT OF INTEREST

The authors declare no conflict of interest.

## Supporting information


Figure S1‐S4

## Data Availability

All datasets generated for this study are included in the article/Supplementary Material.
