# Supplementary material for: Transplantation of Roxadustat‐preconditioned bone marrow stromal cells improves neurological function recovery through enhancing grafted cell survival in ischemic stroke rats
Source: CNS Neurosci Ther. 2022 Jun 13;28(10):1519–31. doi: 10.1111/cns.13890 (PMC9437235; doi:10.1111/cns.13890)
Supplement: Supplementary file 1 — Figure S1‐S4 [file CNS-28-1519-s001.docx]

**Supplementary Material**
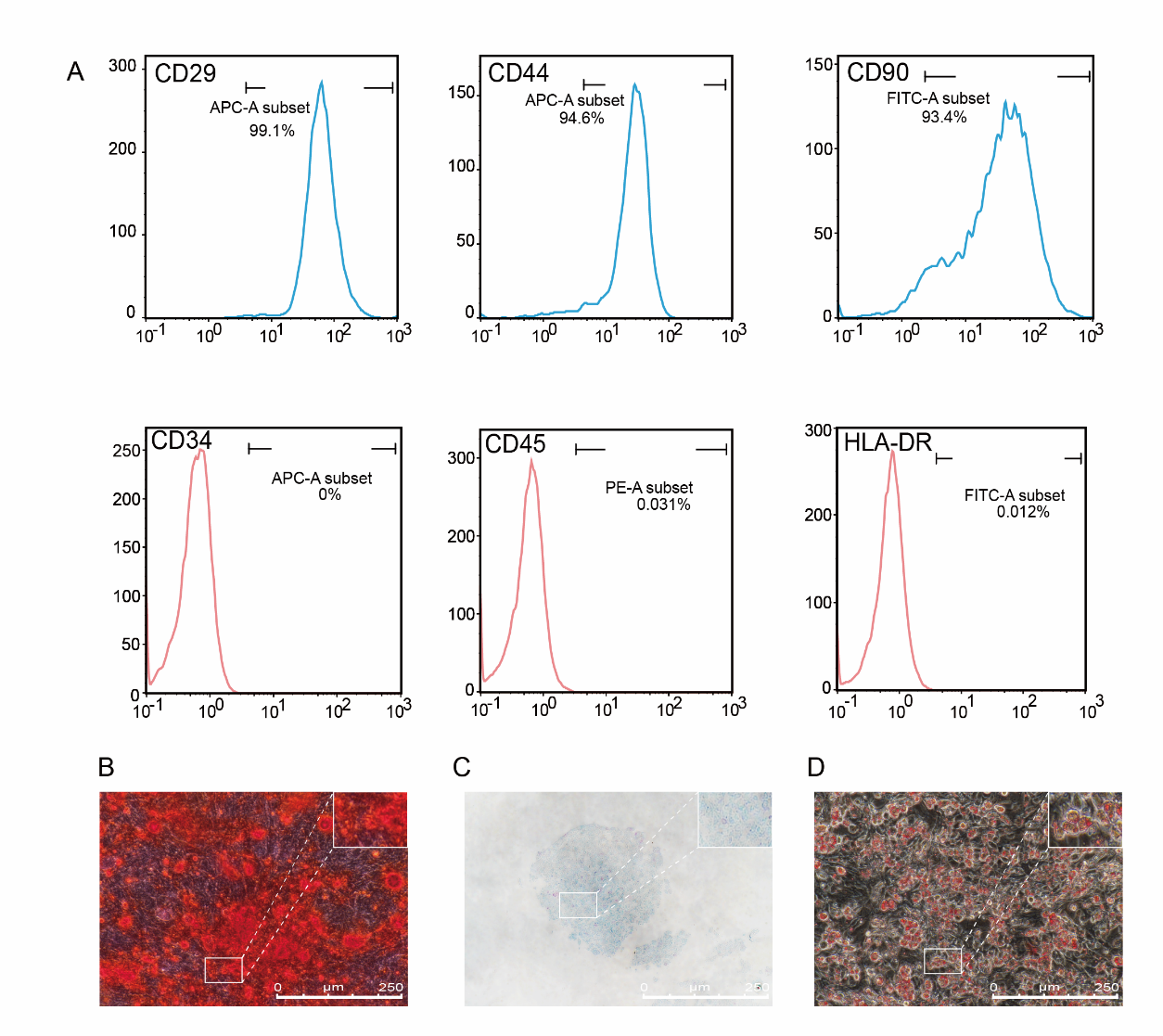


**Figure S1. Identification of BMSCs. (A)** Identification of surface antigens through flow cytometry (CD29, CD44, CD90, CD34, CD45, HLA-DR). **(B)** Differentiation towards osteoblast was monitored by alizarin red staining. **(C)** Cartilage tissue formation was monitored by Alcian blue staining. **(D)** Formation of Adipocytes was monitored by Oil Red O staining. All scale bars=250 µm.


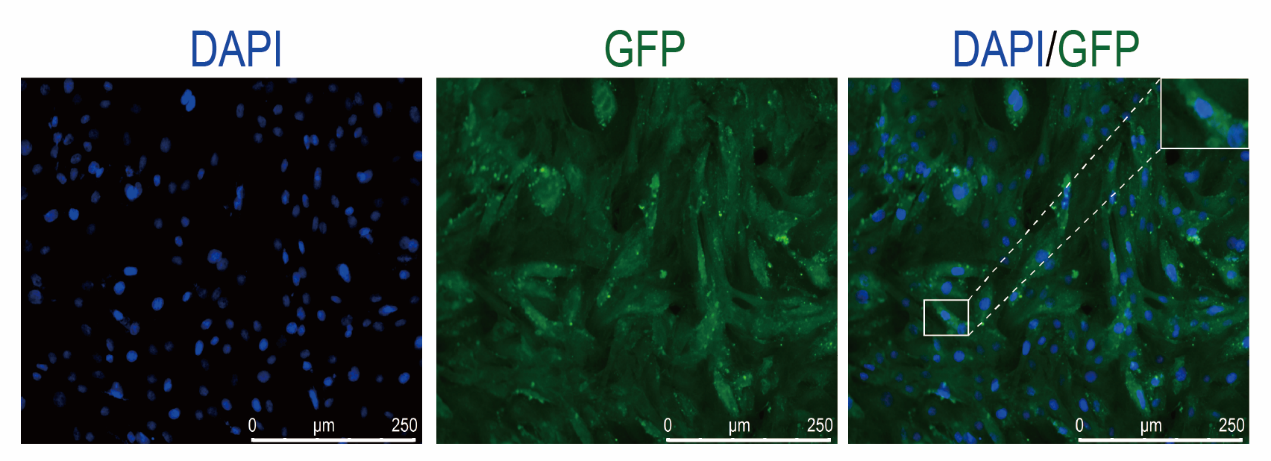
**Figure S2. Representative micrographs of GFP in BMSCs at 3 days after infected with GFP-lentiviruses (n=5). scale bars=250 µm.**

**
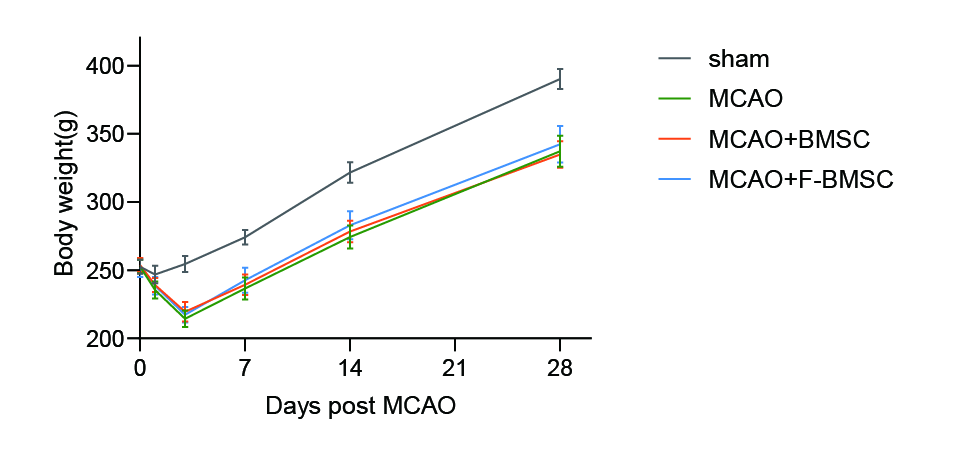
**

**Figure S3. Line chart of body weight after stroke.**

**
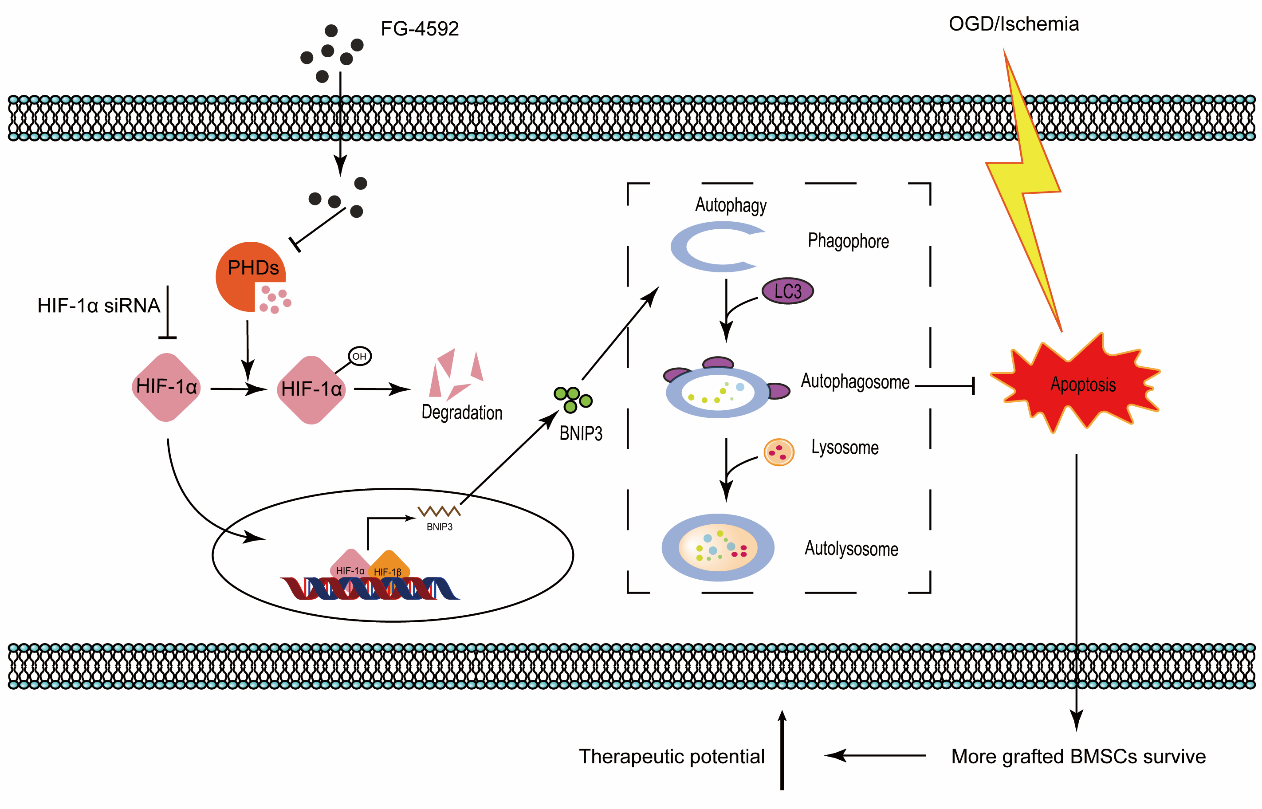
**

**Figure S4. A schematic diagram of the mechanism of FG-4592 enhanced therapeutic capacity of BMSCs.** OGD or ischemia-induced apoptosis of BMSCs. FG-4592-induced activation of HIF-1α/BNIP3 signal pathway, and further activation of autophagy to inhibit apoptosis of BMSCs. Eventually, FG-4592 improves grafted BMSCs survival and enhances therapeutic potential of BMSCs.
